# The different activities of RNA G-quadruplex structures are controlled by flanking sequences

**DOI:** 10.26508/lsa.202101232

**Published:** 2021-11-16

**Authors:** Alice J-L Zheng, Aikaterini Thermou, Pedro Guixens Gallardo, Laurence Malbert-Colas, Chrysoula Daskalogianni, Nathan Vaudiau, Petter Brohagen, Anton Granzhan, Marc Blondel, Marie-Paule Teulade-Fichou, Rodrigo Prado Martins, Robin Fahraeus

**Affiliations:** 1 Inserm UMRS1131, Institut de Génétique Moléculaire, Université Paris 7, Hôpital St. Louis, Paris, France; 2 RECAMO, Masaryk Memorial Cancer Institute, Brno, Czech Republic; 3 Department of Medical Biosciences, Umeå University, Umeå, Sweden; 4 ICCVS, University of Gdańsk, Science, Gdańsk, Poland; 5 ISP, INRAE, Université de Tours, UMR1282, Tours, France; 6 CNRS UMR9187, INSERM U1196, Institut Curie, PSL Research University, Orsay, France; 7 CNRS UMR9187, INSERM U1196, Université Paris Sud, Université Paris-Saclay, Orsay, France; 8 Inserm UMR1078, Université de Bretagne Occidentale (UBO), Etablissement Français du Sang (EFS) Bretagne, CHRU Brest, Brest, France

## Abstract

The role of G-quadruplex (G4) RNA structures is multifaceted and controversial. Here, we have used as a model the EBV-encoded EBNA1 and the Kaposi’s sarcoma-associated herpesvirus (KSHV)-encoded LANA1 mRNAs. We have compared the G4s in these two messages in terms of nucleolin binding, nuclear mRNA retention, and mRNA translation inhibition and their effects on immune evasion. The G4s in the *EBNA1* message are clustered in one repeat sequence and the G4 ligand PhenDH2 prevents all G4-associated activities. The RNA G4s in the *LANA1* message take part in similar multiple mRNA functions but are spread throughout the message. The different G4 activities depend on flanking coding and non-coding sequences and, interestingly, can be separated individually. Together, the results illustrate the multifunctional, dynamic and context-dependent nature of G4 RNAs and highlight the possibility to develop ligands targeting specific RNA G4 functions. The data also suggest a common multifunctional repertoire of viral G4 RNA activities for immune evasion.

## Abstract (precis)

This study demonstrates the dynamic and multifunctional aspects of RNA G4 structures.

## Introduction

RNA–protein interactions are key regulators of the selective processing of RNAs which includes RNA splicing, localisation, translation, and stability, and are therefore involved in various cellular processes (Lewis et al, 2017; Duss et al, 2019; Rodgers & Woodson, 2019) including embryonic development (Beaudoin et al, 2018), neuronal activity (Lin et al, 2020), and oncogenesis (Ceci et al, 2021), to mention some. RNA secondary structures serve as binding platforms for the RNA-binding proteins, determine the assembly of ribonucleoprotein complexes and consequently affect gene expression (Lewis et al, 2017; Beltran et al, 2019; Duss et al, 2019; Rodgers & Woodson, 2019; Sanchez de Groot et al, 2019). This is well illustrated by riboswitches and internal ribosome entry sites (IRESs) that control prokaryotic and eukaryotic/viral gene expression, respectively (Jackson et al, 2010; Serganov & Nudler, 2013). These regulatory elements are usually located in the 5′ UTRs but RNA structures within the coding sequences can also mediate translation. However, little is yet known about the dynamics and regulation of RNA structures in vivo.

G-quadruplexes (G4) are secondary structures found in both RNA and DNA that are formed by the stacking of at least two G-quartets, which are planar arrangements of four guanines connected by Hoogsten hydrogen bonds (Fay et al, 2017; Reznichenko et al, 2019). G4 conformation diversity depends on directionality (parallel, antiparallel, and hybrid), on the number of stacked G-quartets and on the length and sequence of the loops connecting the different strands, affecting stability and binding capacity to specific factors (Harris & Merrick, 2015; Tosoni et al, 2015; Fay et al, 2017). RNA G4 structures are linked to translation suppression when present in 5′ UTR of the mRNA (Beaudoin & Perreault, 2010; Endoh & Sugimoto, 2016; Herdy et al, 2018) and to ribosome stalling when placed in the ORF (Endoh and Sugimoto, 2013, 2016; Endoh et al, 2013a, 2013b). Despite being associated with important functions, the actual formation, stability, and activity of RNA G4s in vivo is still poorly understood and controversial and most of the predicted G4-forming sequences in RNAs do not form stable G4 structures in eukaryotes (Guo & Bartel, 2016). On the other hand, G4 structures in vitro using shorter RNA sequences show high thermostability (Arora & Maiti, 2009).

RNA G4s are implied across various virus families, such as *Flaviviridae* (Fleming et al, 2016; Wang et al, 2016a; Jaubert et al, 2018), *Herpesviridae* (Murat et al, 2014), *Filoviridae* (Wang et al, 2016b), *Paramyxoviridae* (Majee et al, 2020), *Retroviridae* (Ruggiero et al, 2019), and *Coronaviridae* (Bezzi et al, 2021; Ji et al, 2021; Lavigne et al, 2021; Zhao et al, 2021), making them potential targets for drug development. In HIV-1, RNA G4s play an important role in the packaging of the virions (Lyonnais et al, 2003) and are part of the dimerisation process of the two copies of the HIV-1 RNA genome, facilitating recombination during reverse transcription (Marquet et al, 1991, 1994; Piekna-Przybylska et al, 2013; Métifiot et al, 2014). In HCV, RNA G4s are implicated in the translation and replication of the genomic RNA (Wang et al, 2016a; Jaubert et al, 2018). EBNA1 and LANA1 are essential genome maintenance proteins of the EBV and the Kaposi’s Sarcoma–associated herpesvirus (KSHV), respectively (Lan et al, 2004; Münz, 2015). Both are highly antigenic and to allow the viruses to escape immune surveillance the translation of their respective messenger RNAs is kept at the minimum to prevent the production of antigenic peptide substrates for the MHC-I pathway (Yin, 2003; Kwun et al, 2007). The synthesis of antigenic peptides for the MHC-I pathway is via non-canonical translation that is distinct from the canonical translation which generates full-length proteins, in line with the notion that full-length proteins are a poor substrate for antigenic peptides (Cardinaud et al, 2004; Starck et al, 2008; Apcher et al, 2011; Wei et al, 2019; Yewdell et al, 2019). A glycine–alanine repeat (GAr) domain in EBNA1 is encoded by a guanine-rich sequence that forms G4 structures and binds nucleolin (NCL), inhibiting canonical and non-canonical translation initiation *in cis* to support viral immune evasion but how G4 structures support these different activities is not known (Yin, 2003; Apcher et al, 2009; Lista et al, 2017b; Martins et al, 2019). More recently, it was shown that the mRNA translation stress caused by the GAr domain activates the E2F1 oncogene and stimulates c-myc and ribosomal biogenesis (Gnanasundram et al, 2017). Hence, by suppressing its own synthesis, EBNA1 evades the immune system and promotes cell proliferation. The LANA1 of the KSHV also uses a *cis*-acting mechanism to evade immune surveillance (Murat et al, 2014) and as for EBNA1, both RNA G4 structures and repeat domains of LANA1 plays an important role in translation regulation for both full-length proteins and antigenic peptides production (Kwun et al, 2007; Apcher et al, 2009; Murat et al, 2014; Dabral et al, 2019). However, two main differences are worth being noticed when comparing these two functionally similar mRNAs and proteins, whereas the G4s in *EBNA1* are located in the GAr-encoding domain, the G4s of *LANA1* are spread throughout the message (Murat et al, 2014; Dabral et al, 2019). In addition, the peptide repeats of LANA1 and EBNA1 are different, although both are encoded by mRNA sequences predicted to form G4s.

We have used the properties of the *EBNA1* and the *LANA1* mRNAs to study the multifunctional aspects of G4 RNA structures. Each message has different G4 structures encoding different peptide sequences but share functional similarities in suppressing mRNA translation *in cis*, interacting with NCL and preventing mRNA export. The functions of their respective G4s can be separated by alterations in the flanking sequences and together with the observation that RNA G4s can reform in cells, these results illustrate how multifunctional and dynamic G4 structures act together to support viral immune evasion strategies. This sheds new light on some of the controversies surrounding the stability of G4 RNA structures and illustrates the importance of the context in which they are placed.

## Results

### G4 ligands PhenDC3 and PhenDH2 bind LANA1 mRNA G4 structures in vitro

**Figure 1.**
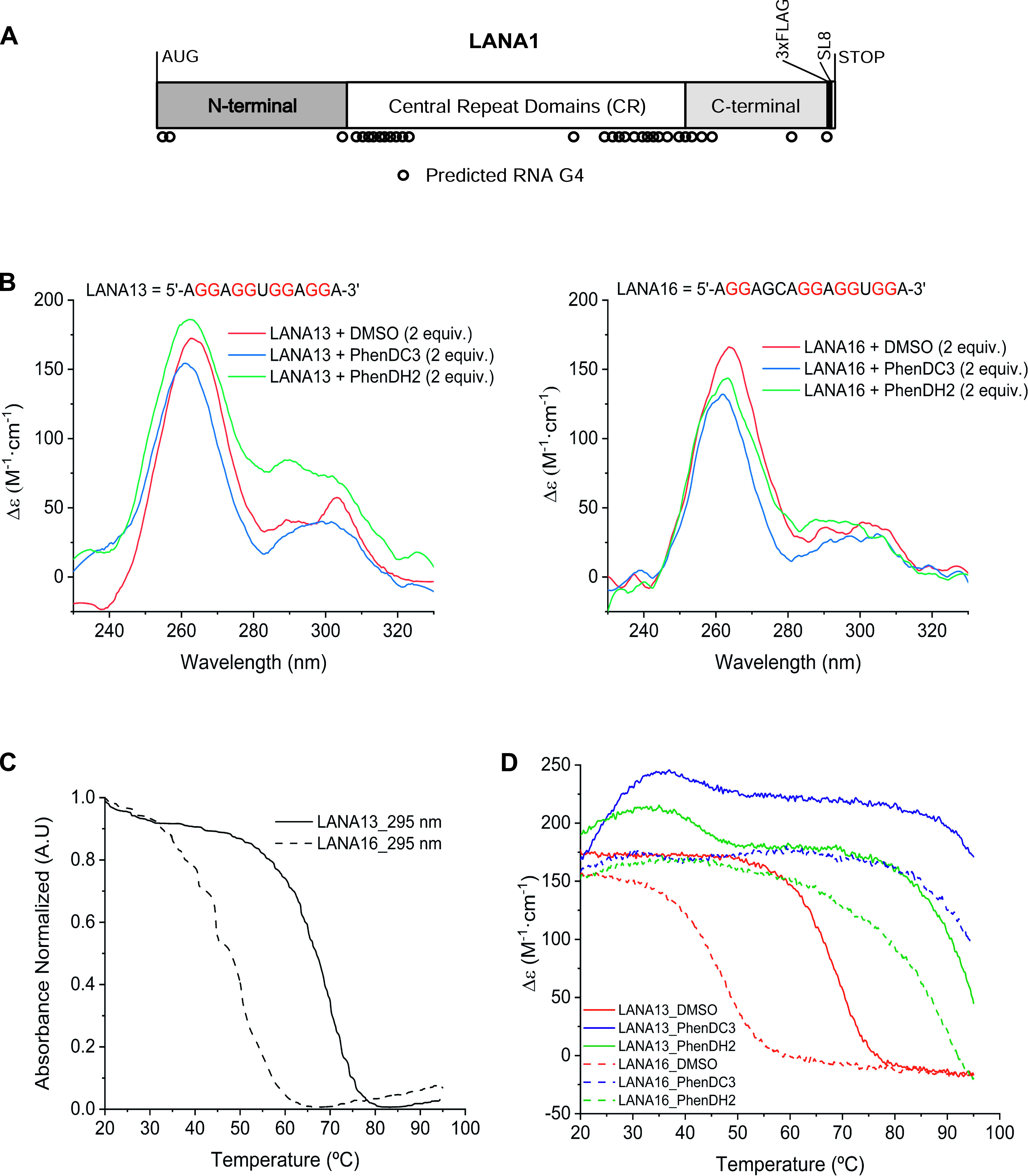
LANA1 central domains encoding mRNA form G4 structures in vitro. **(A)** cDNA construct encoding LANA1 with a FLAG-tag and the SL8 MHC-1 antigenic peptide sequence in its C-terminus. Circles underneath represent the 32 predicted RNA G4 structures. **(B)** Circular dichroism spectra of RNA fragments (5 μM) of LANA1 CR domain in the presence of PhenDC3 (blue), PhenDH2 (green) or without ligand (red). Left, RNA-13mer (LANA13); right, RNA 16-mer (LANA16). **(C)** UV melting experiment at 295 nm of LANA13 (straight line) or LANA16 (dashed line) RNA sequences. **(D)** Circular dichroism melting experiment recorded at 262 nm of LANA13 (straight line) or LANA16 (dashed line) in the presence of PhenDC3 (blue), PhenDH2 (green), or without ligand (red).

**Figure S1.**
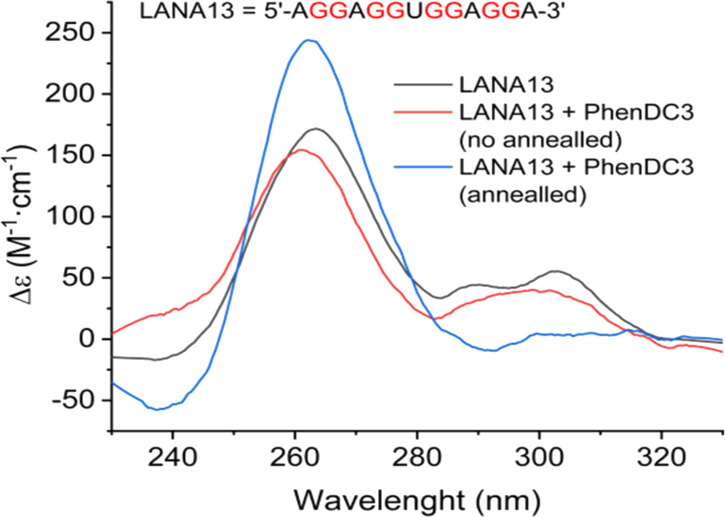
Effects of PhenDC3 ligand on the circular dichroism spectra of the RNA sequence LANA13. RNA (black), in the presence of ligand prior annealing (blue), or after annealing (red). Relates to Fig 1.

Using the QGRS mapper tool, and according to the work of Dabral et al (2019), the LANA1 message can form G4 structure at multiple points on the mRNA, but mostly in the region encoding the Central Repeat Domain of LANA1 (Fig 1A). To confirm the binding of the G4 ligands we selected, namely, PhenDC3 and PhenDH2, on LANA1 RNA G4 structures, two quadruplex-prone G-rich short RNA sequences (LANA13 and LANA16 with QGRS scores of 21 and 18, respectively, Fig 1B) from the LANA1 Central Repeat (CR) domains, which have been already identified for their potential to form multiple quadruplex structures (Dabral et al, 2019), have been selected. First, to experimentally verify the RNA capacity to fold into quadruplex, their circular dichroism (CD) spectra were recorded (Fig 1B). In both cases, a positive peak at 265 nm together with a broad shoulder around 300 nm was observed. The CD spectra indicated the co-existence of different conformations in equilibrium, where the parallel conformation was the predominant. The RNA sequences exhibited a negative transition at 295 nm in the UV melting experiments which is characteristic of G4 unfolding (Fig 1C) and allowed determining Tm values of 65–68°C and 45–48°C for LANA13 and LANA16, respectively. In presence of PhenDC3 or PhenDH2, the CD intensity is slightly decreased indicating that interaction is occurring between the G4 conformation and the ligands (Fig 1B). Interestingly, when PhenDC3 was added before the annealing step, the shoulder at 300 nm was not observed (Fig S1), thereby suggesting that the ligand shifts the equilibrium towards the parallel conformation as has been recently reported for other G4 sequences (Aznauryan et al, 2021). Finally, we monitored the effects of PhenDH2 and PhenDC3 on the RNA sequences by CD melting. PhenDH2 and PhenDC3 induced on LANA16 a very strong stabilization (ΔTm = 40°C for PhenDH2 and >47°C with incomplete melting Tm > 95°C for PhenDC3) (Fig 1D). No complete melting of the G4 of LANA13 could be achieved in the presence of any ligand, indicating a very strong stabilization effect. Taken together, these data support the formation of G4 structures within the LANA1 CR domains and the possibility of targeting them with high affinity ligands such as PhenDC3 and PhenDH2.

### G4 ligands affect *LANA1* mRNA nuclear export, translation, and nucleolin binding

The glycine–alanine repeat (GAr) domain of the EBV-encoded EBNA1 mediates a unique *cis*-acting suppression of its own synthesis via G4 RNA structures to minimise the production of EBNA1-derived antigenic peptides for the MHC-1 pathway (Yin, 2003; Lista et al, 2017b). Similarly, the LANA1 of the KSHV also uses a *cis*-acting mechanism to evade the immune system via its CR domains (Kwun et al, 2007). Whereas the GAr-encoding message consists of GC-rich sequences encoding single alanine residues separated by two or three glycines, the CR domains of *LANA1* are also GC rich, but each repeat region encodes for a different peptide motif (Kwun et al, 2007).

**Figure 2.**
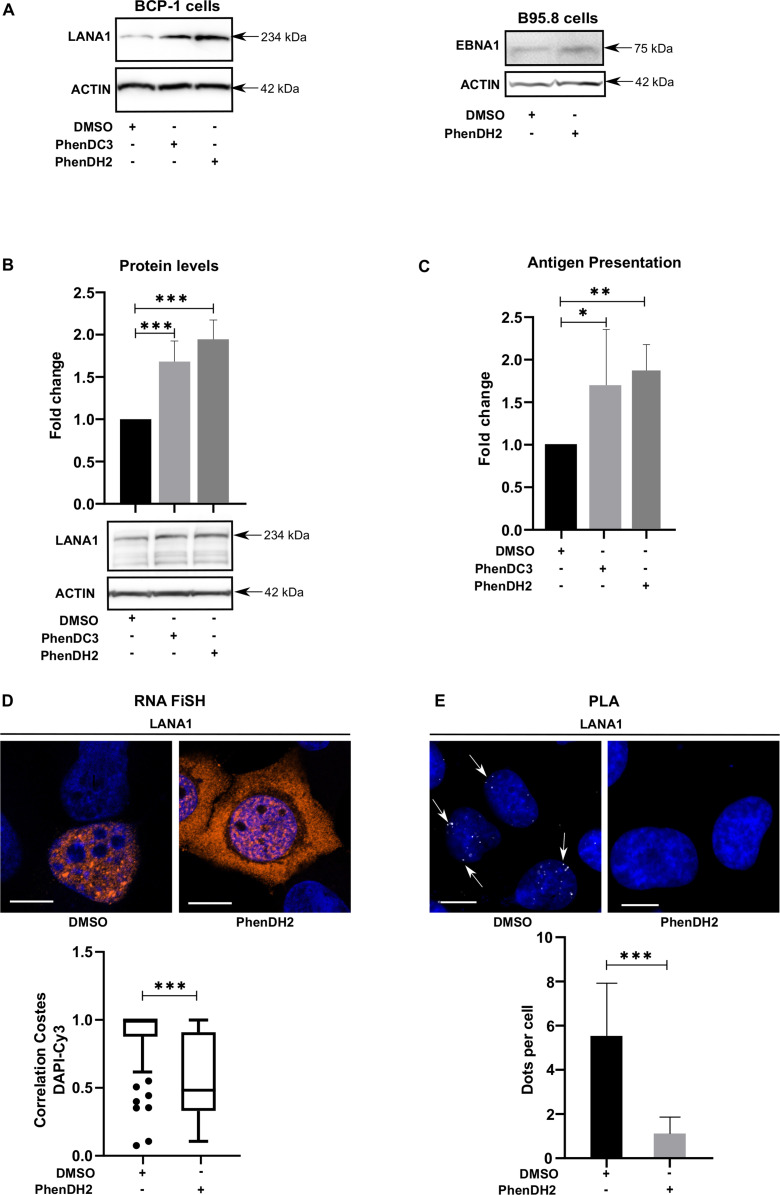
G4 RNA structures control the translation and processing of the *LANA1* mRNA. **(A)** Western blots (WB) show the expression of LANA1 in KSHV-infected BCP-1 cells (left) or EBNA1 in EBV-infected B95-8 cells (right) after treatment with 2 μM of G4 ligands PhenDC3, PhenDH2, or DMSO for 24 h. WB show one of at least three similar experiments. **(B)** WB (below) shows the expression of LANA1 from cDNA construct presented in Fig 1A after indicated treatments. The graph above shows expression relative to actin from three independent experiments. WB shows one of at least three similar experiments. **(C)** Relative amount of SL8 antigen peptide produced from the LANA1 cDNA presented in Fig 1A under similar conditions in H1299 cells expressing the murine MHC-I (Kb). The levels were estimated by measuring IL2 release from OT1 CD8^+^ T cells. **(D)** *LANA1* mRNA localisation (RNA FiSH) in H1299 cells transfected with the LANA1 construct presented in Fig 1A after PhenDH2 or DMSO treatment. The boxplot graph below shows Costes Correlation factor for cytoplasmic versus nuclear localisation. **(E)** The proximity ligation assay shows the interaction between the *LANA1* mRNA and nucleolin (NCL) (white dots) after PhenDH2 treatment. The graph below shows the average interactions per cell from three independent experiments. Scale bar corresponds to 10 μm.

To know if *LANA1* and *EBNA1* are using similar molecular mechanisms involving RNA G4 structures to control mRNA translation, we first treated KSHV-carrying lymphoma B cells (BCP-1) and the EBV-carrying B-cell line B95.8 with 2 μM of the G4 ligands PhenDC3 or PhenDH2 for 24 h (Reznichenko et al, 2019). This resulted in an increase in the expression of respective proteins (Fig 2A). For EBNA1, this is linked to prevention of NCL binding to the RNA G4s by the G4 ligands (Lista et al, 2017a, 2017b). Thus, this result shows that G4 structures in the *LANA1* message, like in the *EBNA1* message, are involved in translation suppression.

**Figure S2.**
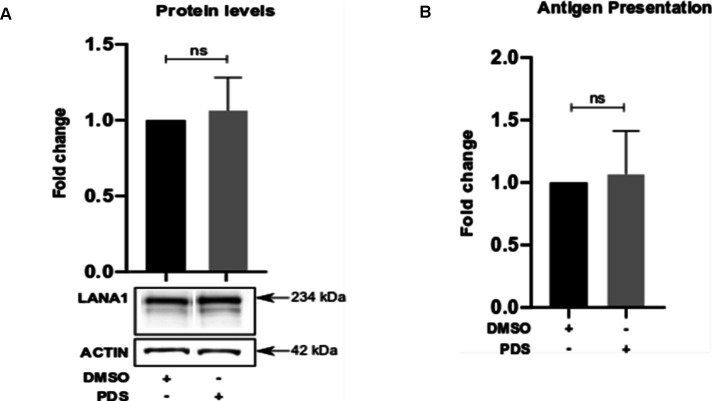
Effects of the G4 RNA PDS ligand. **(A)** Western blots (WB) show the expression of LANA1 from cDNA construct presented in (Fig 1A) the following treatment with 2 μM of G4 ligands PDS for 24 h. WB show one of at least three similar experiments. **(B)** Relative amount of SL8 antigen peptide produced from the LANA1 cDNA presented in (Fig 1A) under similar conditions in H1299 cells expressing the murine MHC-I (Kb). The levels were estimated by measuring IL2 release from OT1 CD8^+^ T cells. Relates to Fig 2B and C.

We next expressed a FLAG-tagged LANA1 construct (Fig 1A) in human H1299 carcinoma-derived cells and we observed a similar increase in protein levels after PhenDC3 and PhenDH2 treatment (Fig 2B), showing that the G4-mediated translation control is not dependent on cell line or on the presence of the whole viral genome, as for EBNA1 (Lista et al, 2017b). Unlike PhenDC3 and PhenDH2, another G4 ligand, the pyridostatin (PDS), is not able to interfere with the inhibition of LANA1 expression (Fig S2A), similarly to what was shown with EBNA1 (Lista et al, 2017b). We also inserted an eight amino acid–MHC-I antigenic peptide sequence (SL8) derived from the chicken ovalbumin gene (Ova) in the C-terminus of LANA1. The SL8 peptide is specifically recognised by CD8^+^ T cells (OT-1) when presented on the murine MHC-I (Kb) (Martins et al, 2019). After transient expression of LANA1 together with Kb in H1299 cells, we observed a twofold activation of CD8^+^ T cells after G4 ligand treatment, meaning that more SL8 antigenic peptide substrates were synthesised (Fig 2C), whereas the PDS is not able to interfere with the production of antigenic peptides for MCH-I pathway (Fig S2B). As PhenDH2 is a more potent G4 ligand when it comes to *EBNA1* mRNA compared with PhenDC3 (Reznichenko et al, 2019), we focused our investigation on PhenDH2 treatment. By using RNA FiSH, we observed that the *LANA1* mRNA is located in the nucleus and that treatment with PhenDH2 promoted a cytoplasmic location (Fig 2D). Furthermore, using the proximity ligation assay (PLA) we showed that PhenDH2 interferes with the *LANA1* mRNA–nucleolin (NCL) interaction in situ (Fig 2E).

These results show that the EBV and KSHV have evolved a common strategy to exploit *cis*-acting G4 mRNA structures in the *EBNA1* and *LANA1* messages to prevent the production of antigenic peptide substrates for the MHC-1 pathway.

### Deleting the LANA1 CR domain overcomes nuclear retention and stimulates synthesis of antigenic peptide substrates but does not affect the synthesis of full-length proteins or the interaction with nucleolin

**Figure 3.**
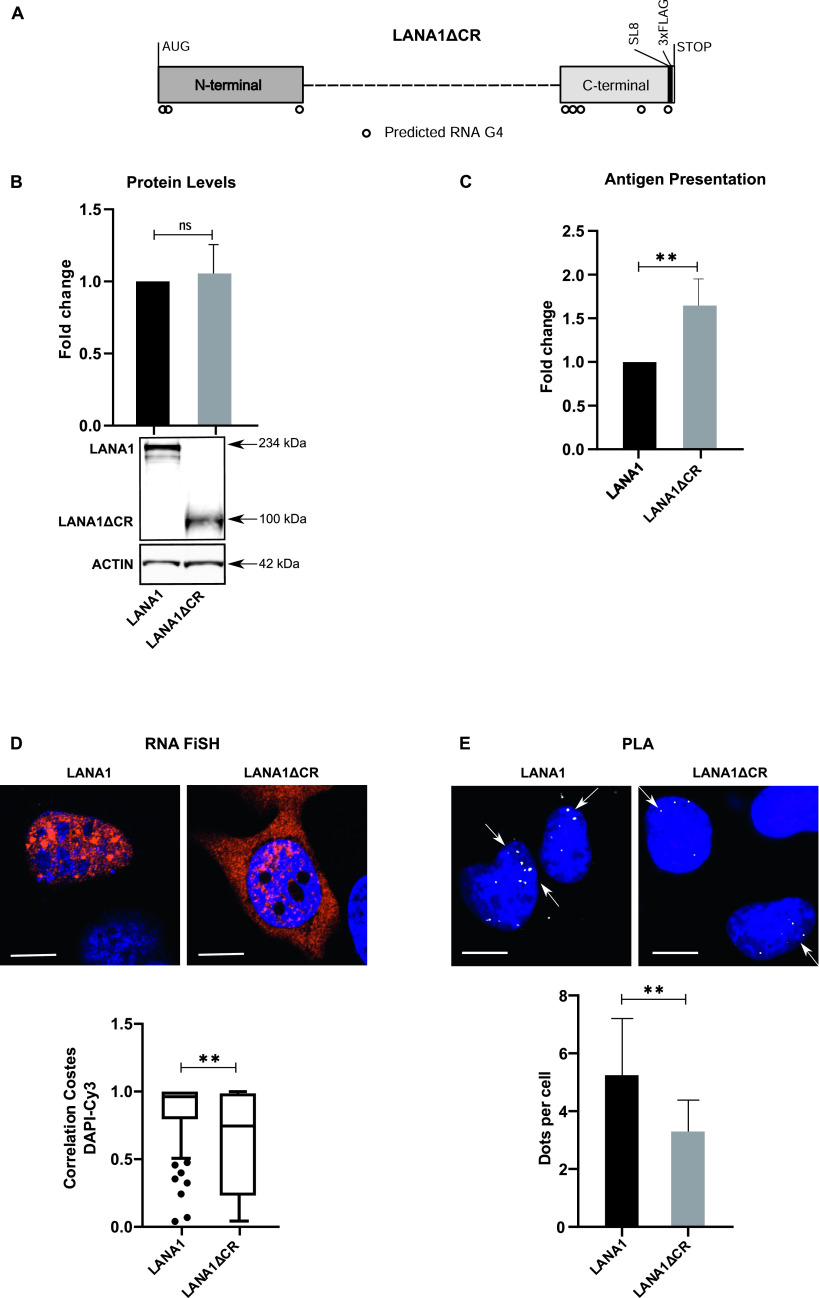
Central repeat (CR) domains of the *LANA1* mRNA control antigen presentation, nuclear retention but not canonical translation for production of full-length protein or nucleolin interaction. **(A)** cDNA construct encoding LANA1 without CR domains (LANA1ΔCR) and with a FLAG Tag and the SL8 in the C terminus. Circles underneath the rectangles represent the eight predicted RNA G4 structures. **(B)** WB of H1299 cells transfected with the LANA1 or LANA1ΔCR constructs. Graph shows relative protein levels, adjusted to corresponding actin control. WB shows one of at least three similar experiments. **(C)** Relative antigen presentation levels estimated by measuring IL2 release by OT1 cells after incubation with H1299 cells expressing murine MHC-I (Kb). **(D)** *LANA1* mRNA localisation (RNA FiSH) with boxplot graph below representing three independent experiments. **(E)** The interaction with NCL and the *LANA1* mRNA (PLA NCL-*LANA1* mRNA) in H1299 cells transfected with indicated cDNA constructs. The graph below shows the average of three independent experiments. Scale bar corresponds to 10 μm.

It has been suggested that the CR domains of the *LANA1* account for mRNA translation control (Kwun et al, 2007) and we wanted to know if G4 activity in this region mediates the same functions as the EBNA1-encoded *GAr* sequence. When we deleted the CR domains (LANA1ΔCR), we observed no, or little, change in LANA1 protein expression, whereas we observed an average 60% increase in antigen presentation, indicating that different G4-forming domains in *LANA1* control translation of antigenic peptide substrates and full-length proteins (Fig 3A–C). RNA FISH analysis detected most *LANA1ΔCR* messages in the cytoplasm (Fig 3D). The PLA showed NCL–*LANA1ΔCR* mRNA interactions in the nucleus, even though the number of interactions were reduced when compared with full-length *LANA1* (Fig 3E). It should be pointed out that NCL is nuclear and preventing nuclear retention of the mRNAs could be sufficient to explain the differences in number of interactions.

Thus, G4 structures of the CR domain of the *LANA1* message mediate nuclear retention and control of antigenic peptide synthesis but not full-length protein synthesis and NCL interaction.

### G4s outside the CR domains control LANA1 protein expression and nucleolin binding

**Figure 4.**
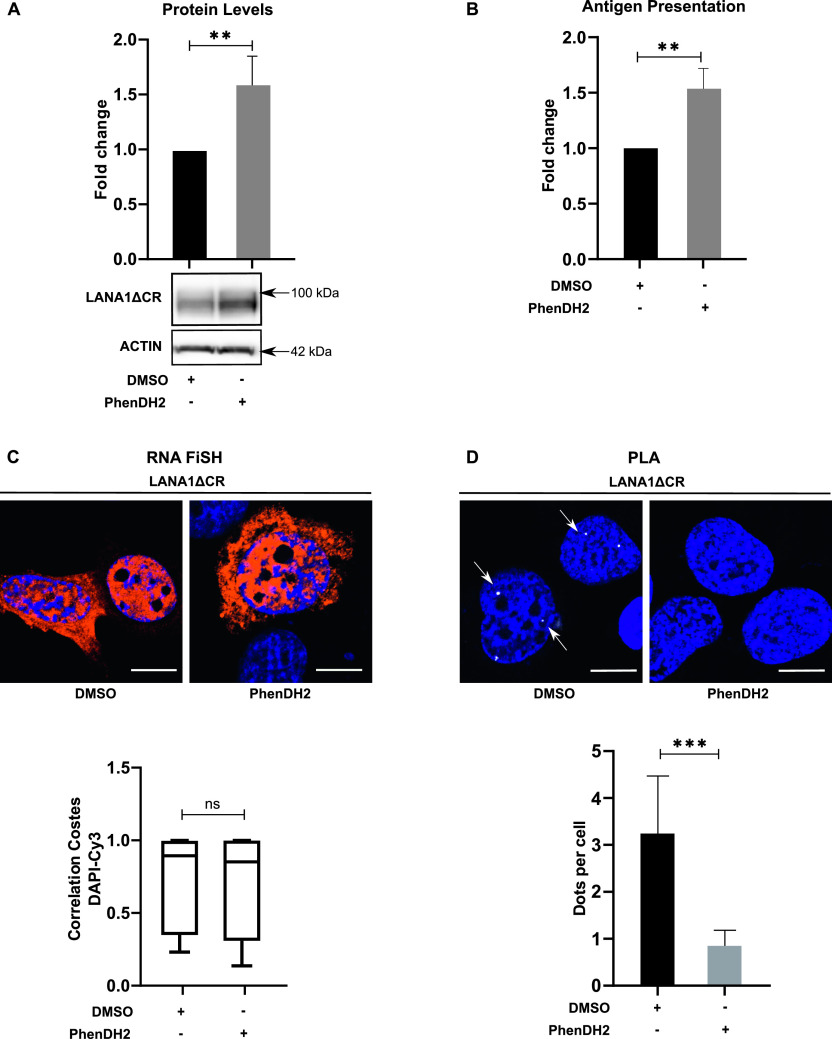
G4 structures outside the central repeat (CR) of *LANA1* control NCL binding and canonical translation. **(A)** WB from H1299 cells expressing LANA1ΔCR treated with the G4 ligands PhenDH2 or DMSO. Graph shows relative protein levels adjusted with the corresponding actin levels. WB shows one of at least three similar experiments. **(B)** Relative antigen presentation estimated by measuring IL2 release by OT1 cells after incubation with H1299 cells expressing murine MHC-I (Kb) and LANA1ΔCR and treated with PhenDH2 or DMSO. **(C)** *LANA1ΔCR* mRNA localisation (RNA FiSH). **(D)** Proximity ligation assay showing the RNA–NCL interaction (proximity ligation assay NCL-*LANA1* mRNA) in H1299 cells transfected with LANA1ΔCR construct after PhenDH2 or DMSO treatment. The graphs show the average of three independent experiments. Scale bar represents 10 μm.

The above results indicate that different *LANA1* mRNA processing activities can be mediated by individual G4 structures throughout the *LANA1* message. To test if NCL binding and the control of full-length protein synthesis of the LANA1ΔCR construct is mediated by G4s predicted outside the CR domain, we treated cells expressing LANA1ΔCR with PhenDH2. This resulted in an average 50% increase in protein levels (Fig 4A) and antigen presentation (Fig 4B). The treatment did not significantly affect *LANA1ΔCR* mRNA localisation (Fig 4C) but prevented furthermore the interaction with NCL (Fig 4D).

This confirms that the *cis*-mediated regulation of *LANA1* mRNA translation is more complex as compared with the *EBNA1* and involves G4s from different regions of the coding sequence and not just from the CR domains.

### The position of the G4s within the coding sequence controls its activity

**Figure 5.**
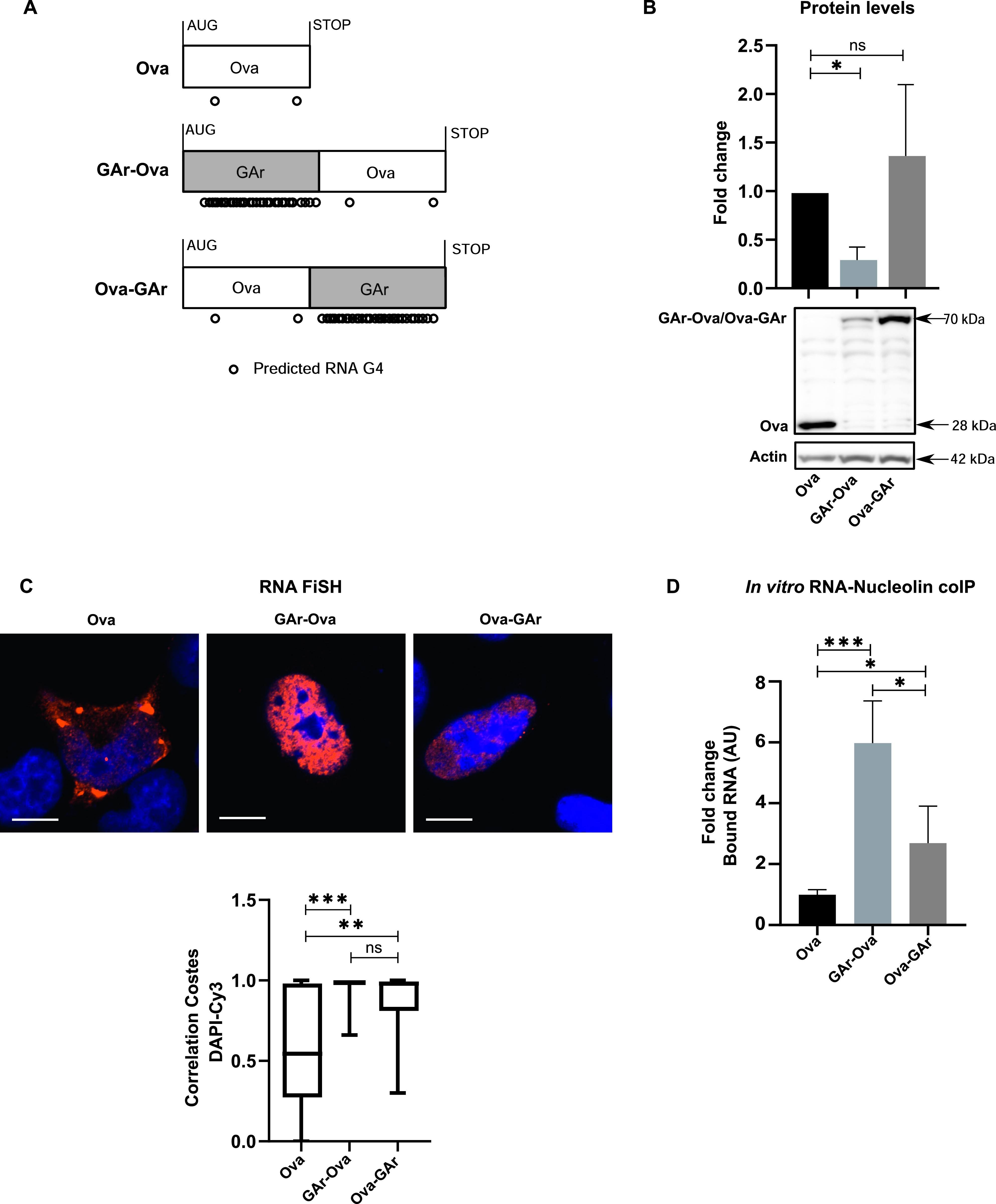
The position of G4s within the coding sequence affects their respective activities. **(A)** Illustrations of the cDNA constructs to which GAr was fused to the N terminus (GAr-Ova) or C terminus (Ova-GAr) of chicken ovalbumin (Ova). Circles underneath represent the predicted RNA G4 structures for each construct (2 for Ova, 34 for GAr-Ova, and 33 for Ova-GAr). **(B)** Western blot of H1299 cells after transfection with indicated cDNA constructs. Graph shows relative expression adjusted with the corresponding actin levels. WB shows one of at least three similar experiments. **(C)** Ova-encoding mRNA localisation (RNA FiSH) in H1299 cells transfected with indicated constructs. Scale bar represents 10 μm. **(D)** Relative binding of Ova fusion mRNAs to recombinant NCL protein measured by in vitro RNA co-IP assay. mRNAs were extracted from H1299 cells transfected with indicated constructs. The graphs show the average of three independent experiments.

**Figure S3.**
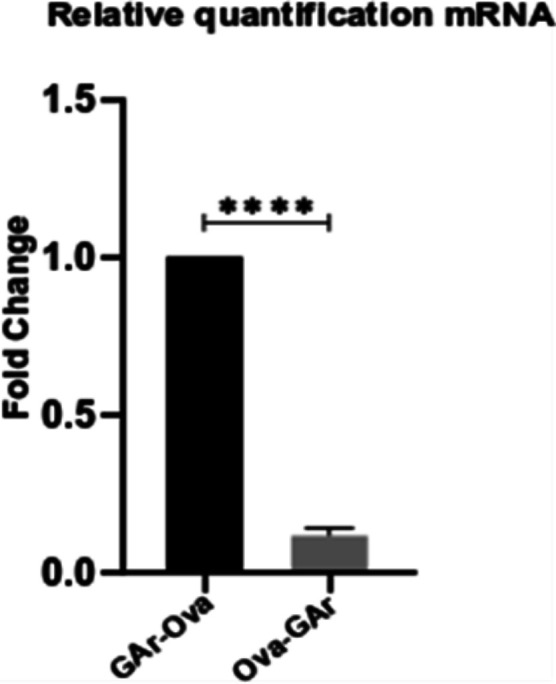
mRNAs encoding GAr fused to Ova relative levels 24 h after transfection of H1299 cells with the indicated constructs. Relates to Fig 5.

It has been suggested that the interaction between NCL and G4s of *EBNA1* mRNA is sufficient for suppressing synthesis of antigenic peptides and full-length proteins (Lista et al, 2017b), and it was surprising to see that deletion of the CR differentiates these two activities in the *LANA1* mRNA. Therefore, we were interested to know if the G4 activities of the *EBNA1* message can also be separated. To test this, we used constructs in which the GAr was fused to the 5′ or the 3′ end of the chicken ovalbumin (Ova) ORF, resulting in fusion proteins with the GAr in the N terminus or the C terminus (GAr-Ova and Ova-GAr, respectively) (Fig 5A) (Yin, 2003). Both messages retained their nuclear localisation but the expression of the GAr-Ova fusion protein was considerable less, as compared with the Ova-GAr, whereas the RNA levels followed an opposite trend (Figs 5B and C and S3). When we isolated these RNAs from transiently transfected cells and compared their binding capacity to recombinant NCL, we observed that the *GAr-Ova* mRNA had a higher affinity to NCL compared with the *Ova-GAr* mRNA (Fig 5D).

Hence, the location of the GAr G4s within the coding sequence of the message determines NCL binding and mRNA translation inhibitory activity but not nuclear retention. Of note, these results support the notion that NCL plays no role in G4-dependent mRNA export.

### Flanking UTR sequences affect *LANA1* and *EBNA1* G4s differently

**Figure 6.**
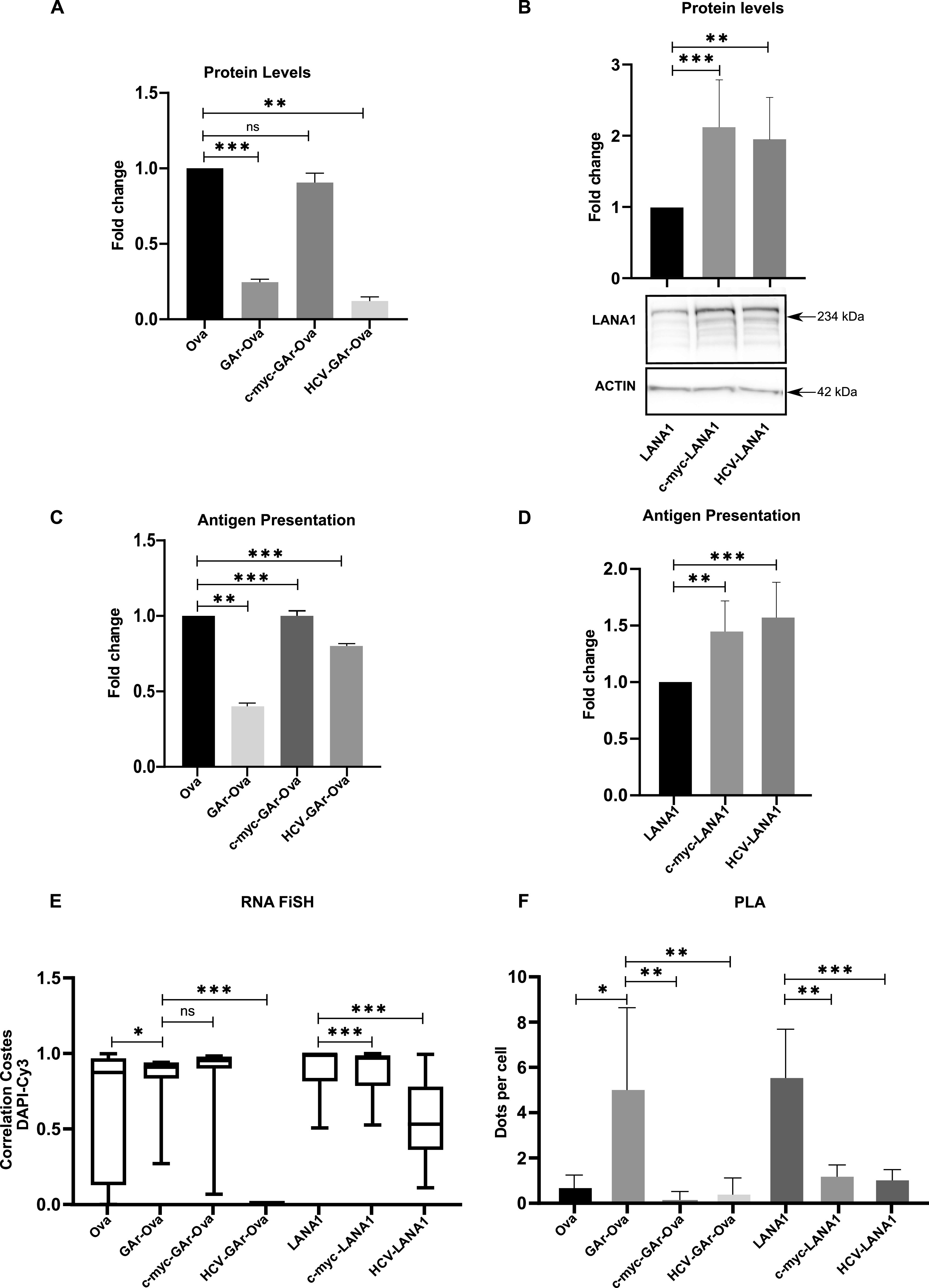
Altering the UTRs differentiates the activities of the GAr-encoded and *LANA1* G4s. Structured RNA sequences from the 5′ of the *c-myc* and the *HCV* were inserted in the 5′ of indicated constructs. **(A)** WB of H1299 cell lysates after transfection with indicated Ova fusion constructs. GAPDH was used as loading control. **(B)** WB of H1299 cell lysates after transfection with indicated LANA1 fusion constructs. **(C)** Antigen presentation levels estimated by measuring IL2 release by OT1 cells after incubation with H1299 cells transfected with GAr fusion constructs together with cDNA encoding murine MHC-I (Kb). **(D)** Antigen presentation levels derived from LANA1 fusion constructs. **(E)** Graph from RNA FiSH observations shows the median subcellular colocalisation of indicated mRNAs with the nucleus. **(F)** Graph shows proximity ligation assay data about the interaction between NCL and indicated mRNAs from transfected constructs in H1299 cells. The graphs show the values of three independent experiments.

**Figure S4.**
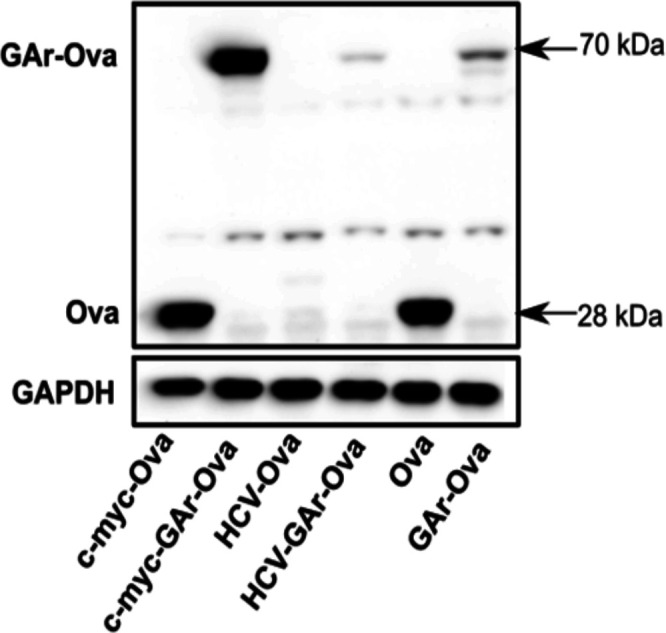
Western blot with cell lysate from H1299 cells transfected with Ova/GAr-Ova constructs bearing c-myc or HCV IRES in the 5′UTR. Relates to Fig 6.

**Figure S5.**
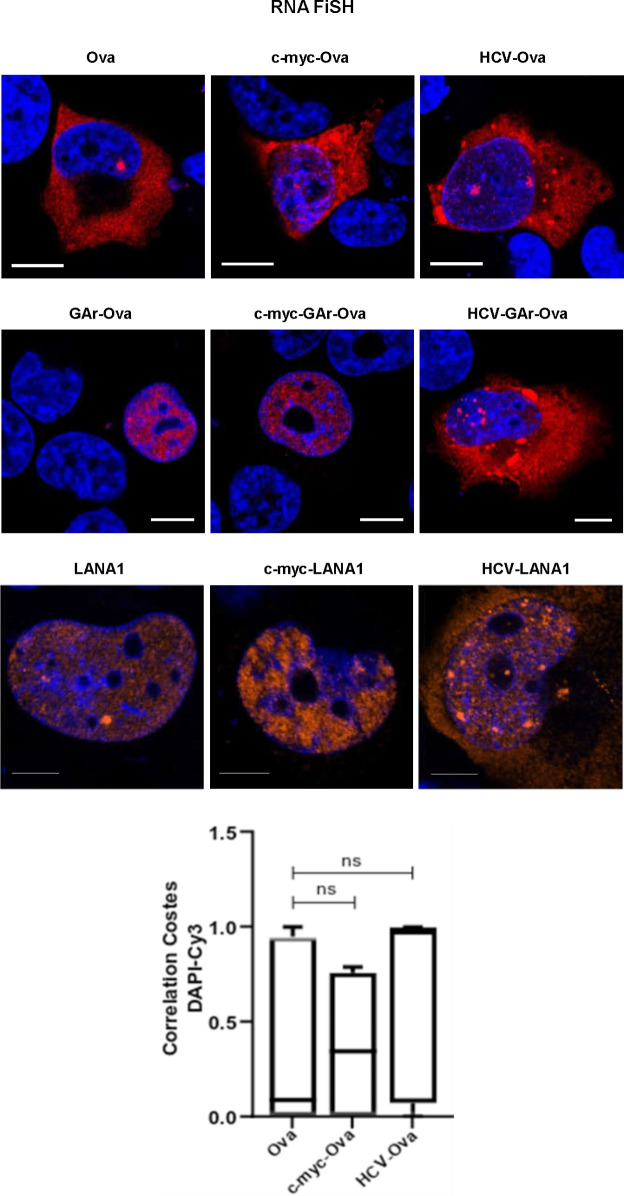
Ova (upper panels) and LANA1 (lower panels) mRNA localisation (RNA FiSH) in H1299 cells transfected with the indicated constructs (scale bar: 10 μm). The graph below shows Costes Correlation factor between nucleus and LANA1 mRNA staining in H1299 cells transfected with the indicated cDNA. Relates to Fig 6E.

**Figure S6.**
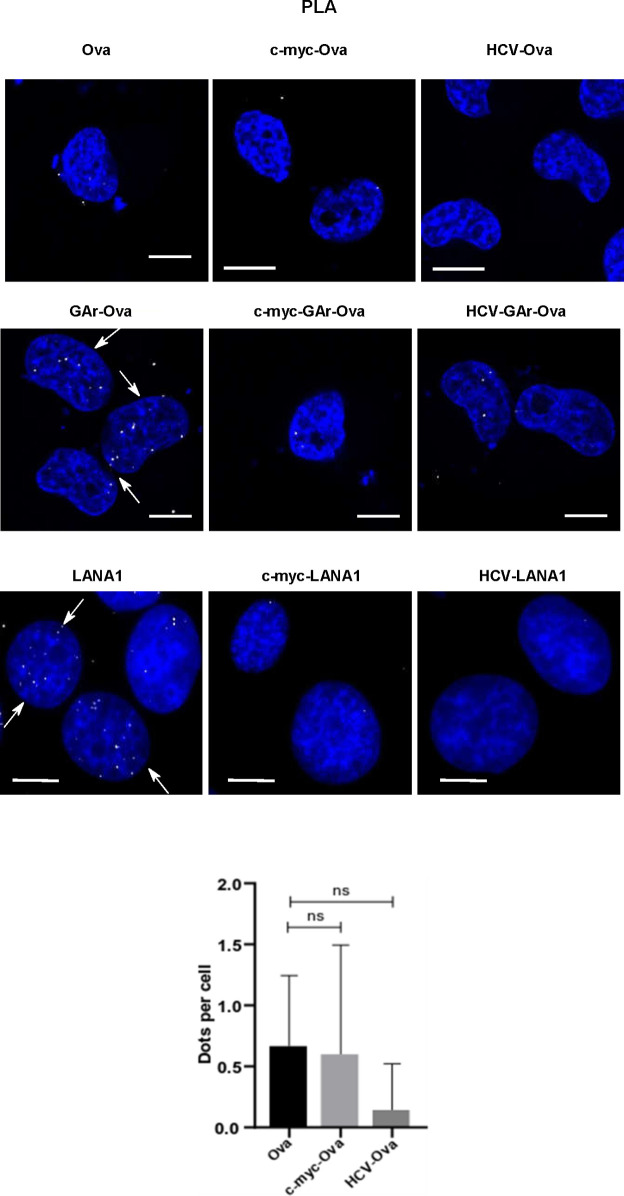
Proximity ligation assay for screening nucleolin-Ova mRNA (upper panels) and nucleolin-LANA1 mRNA (lower panels) in H1299 cells transfected with the indicated constructs (scale bar: 10 μm). The graph below shows the number of dots per cells resulting from the proximity ligation assay between NCL protein and Ova mRNA Relates to Fig 6F.

**Figure S7.**
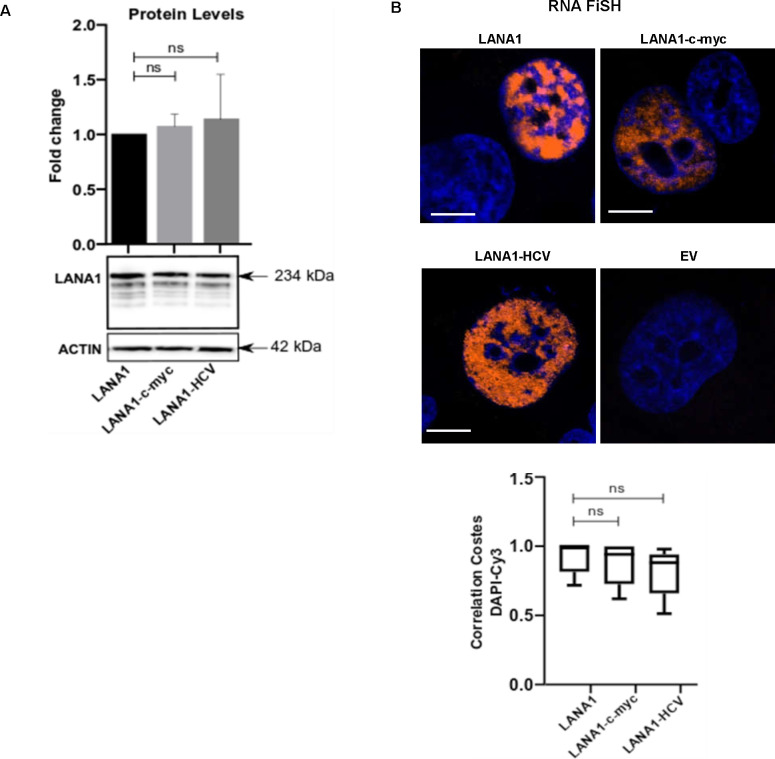
Altering the 3′ UTR does not affect the activities of the *LANA1* G4s. **(A)** Western blot with cell lysate from H1299 cells transfected with LANA1 FL constructs bearing c-myc or HCV IRES in the 3′ UTR. The graph above shows expression relative to actin from three independent experiments. **(B)** LANA1 mRNA localisation (RNA FiSH) in H1299 cells transfected with cDNA constructs as in (A) (scale bar: 10 μm). The graph below shows Costes Correlation factor between nucleus and LANA1 mRNA staining . Relates to Fig 6.

The GAr-Ova and the Ova-GAr reporter constructs showed that the localisation of the G4-containing translational inhibitory sequence within the coding sequence is important for some, but not all, G4-related activities. We next wanted to know if changes in the non-coding sequences can also affect G4 functions. We introduced RNA structures in the form of the *c-myc* and *HCV* IRESs in the 5′ UTR of the GAr-Ova construct. We could confirm that the *c-myc*-GAr-Ova construct and *c-myc*-Ova are equally efficiently translated (Apcher et al, 2009). The fusion of the *HCV* IRES (*HCV*-GAr-Ova) instead further suppressed expression of GAr-Ova as well as Ova alone (*HCV*-Ova) (Fig 6A) (Martins et al, 2019). When the two IRESs were individually fused to the *LANA1* message, they both stimulated synthesis of full-length proteins (Fig 6B). Interestingly, although only the *HCV* IRES suppressed synthesis of GAr-Ova and Ova proteins, the presence of either IRES in the 5′ UTRs resulted in an increase in the synthesis of antigenic peptide substrates from *GAr-Ova* and *LANA1* messages (Fig 6C and D). Hence, although the *HCV* IRES suppressed expression of full-length GAr-Ova and Ova, it stimulated synthesis of antigenic peptide substrates from both *GAr-Ova* and *LANA1* messages. Both IRESs also prevented NCL binding to GAr-encoding and *LANA1* mRNAs, but only the *HCV* IRES promoted mRNA nuclear export (Figs 6E and S4–S6). Fusing the *c-myc* and the *HCV* structures to the 3′ UTR of *LANA1* had little effects on G4 activities (Fig S7A and B).

This demonstrates that the different functions of the G4s within the messages can be separated by altering the 5′ UTR. These data further underline that the synthesis of full-length proteins and antigenic peptides are derived from two independent mRNA translation events.

## Discussion

We have compared four different activities related to G4 structures within the coding sequences of the KSHV- and EBV-encoded LANA1 and EBNA1, respectively. These proteins play similar key roles in maintaining viral genome integrity and both are expressed in all virus-infected cells, including cancerous ones, making their respective expression interesting therapeutic targets for virus-associated diseases (Ballestas, 1999; Ballestas & Kaye, 2001; Ciufo et al, 2001; Kwun et al, 2007; Münz, 2015). Both viruses need to ensure that the production of peptide substrates for the MHC-I pathway is kept at a minimum to evade the immune system (Kwun et al, 2007; Murat et al, 2014). To achieve this, they have evolved *cis*-acting mechanisms to suppress translation of the *LANA1* and *EBNA1* messages. Even though the peptide sequences mediating this effect are not similar (Kwun et al, 2007), the concept to control mRNA processing and translation to achieve immune evasion is. This suggests that controlling their own rate of translation might be a common strategy whereby, at least latent, viral messages evade the immune system. In agreement with this hypothesis is the observation that viruses causing persistent infections in *Metazoa* hosts are significantly enriched with putative G-quadruplex forming sequences (PQS), whereas viruses causing acute infections are significantly depleted in PQS (Bohálová et al, 2021).

Treatment with the G4 ligand PhenDH2 resulted in an increase in protein expression, antigen presentation, nuclear export, and loss of NCL binding in the context of EBNA1 and LANA1, indicating that these four events are indeed all G4 dependent. The G4s of *EBNA1* mRNA are mostly clustered within the gly–ala repeat GAr-encoding sequence (Murat et al, 2014), whereas the G4 activities of *LANA1* are spread throughout the message. The G4s in the CRs of *LANA1* are determinant for nuclear mRNA retention and suppression of antigenic peptide substrates, whereas G4s outside the CR domains are important for canonical translation control and nucleolin interaction. Both CR and non-CR–related G4s play roles in antigen production. The fact that the CR domains play an important role in controlling the synthesis of antigenic peptides is in line with previous observations (Kwun et al, 2007). However, the results presented here do not support the CR domains being the sole responsible for controlling synthesis of antigenic peptides and full-length proteins from the *LANA1* mRNA. Nevertheless, we did not analyse the role of the individual LANA1 repeat domains and together with the fact that flanking sequences play a role in determining G4 activities this can help in explaining this discrepancy.

An important conclusion from this study is that different G4 activities within an mRNA can be differentiated by altering flanking sequences, illustrating the dynamic nature of G4 RNA structures. For example, moving the GAr sequence from the 5′ towards the 3′ end of the coding sequence reduces its translation inhibitory capacity and NCL binding without affecting nuclear retention. Introducing the structured RNAs of the *HCV* or the *c-myc* IRESs affect *LANA1* and *GAr* mRNA translation and localisation differently. The *HCV* IRES further suppressed synthesis of GAr-carrying mRNAs, whereas it instead stimulated LANA1 expression and released the nuclear retention of both mRNAs. The *c-myc* IRES, on the other hand, does not affect RNA localisation but induces translation of both messages (Martins et al, 2019). Also G4-mediated regulation of canonical translation producing full-length proteins and non-canonical translation linked to antigenic peptides synthesis could be separated by introducing the *HCV* IRES in the 5′ of the *GAr*.

Previous works show that RNA G4 structures can fold, unfold and refold *in cellulo* (Biffi et al, 2014; Chen et al, 2018; Yang et al, 2018). The *EBNA1* message does not contain introns but placing the GAr in the context of a gene prevents NCL access *in cellulo*. However, placing the GAr in this context does not affect the G4 structure and RNAs isolated from cells interact with recombinant NCL (Martins et al, 2019). The interaction between the *GAr* derived from a spliced construct and NCL was observed in the cytoplasm when the nuclear localisation signal of NCL was removed (Martins et al, 2019). As the spliced message is subjected to the pioneer round of translation, the GAr G4s would have had to refold to allow NCL interaction in the cytoplasm. These observations indicate that the function of G4s in the regulation of mRNA processing is strongly influenced by events taking place during the mRNA maturation process and that they can act both in the nucleus as well as in the cytoplasm. In this context, G4s multifunctionality might be affected by any change impacting the nature of the ribonucleoparticles. In fact, several RNA binding proteins are reported to be involved in the folding and unfolding of G4s RNA for a specific regulation event (Wolfe et al, 2014; Herviou et al, 2020; Vannutelli et al, 2020).

The role, and actual in vivo existence, of G4 RNA structures is controversial (Guo & Bartel, 2016; Waldron et al, 2018; Dumas et al, 2021). Our data support a model in which G4 structures form dynamic multifunctional units, showing similarity to the concept of intrinsic disordered proteins. The allosteric conformation of intrinsic disordered proteins is regulated by post-translational modifications and/or ligand binding that alter their interactomes and their functions (Habchi et al, 2014; Borkosky et al, 2017). The RNA G4s could follow a similar scheme, in which ligand binding, or RNA modifications, could affect the structure of the G4s and alter their activity. In the examples described here, the activities of the *LANA1* and *EBNA1* G4s can be disrupted by chemical ligands, by moving their position or by changing flanking sequences.

It is conceivable that the dynamic multifunctional roles of these herpes viral RNA G4s are not unique but reflect a more common nature of regulated RNA structures within coding sequences. In the case of the *p53* mRNA, a structured region in the 5′ of the coding sequence is folded during genotoxic stress so that it binds to MDM2 and stimulates p53 synthesis. The RNA folding is prevented by a single cancer-derived synonymous mutation, illustrating how single nucleotides can affect the folding of an mRNA and its interaction with regulatory proteins (Candeias et al, 2008; Naski et al, 2009). In line with this, modifications of mRNAs can result in structural changes and, consequently, affect their functions and interactions with cellular factors (Lewis et al, 2017). For example, the binding of hnRNPC to cryptic U-tract sequences can be made available via *N6*-methylation on adenosine residues (Liu et al, 2015).

Treatment with chemical G4 ligands PhenDC3 or PhenDH2 disrupts *GAr* and *LANA1* G4s functions. However, the PDS G4 ligand stimulates the inhibition of EBNA1 synthesis in vitro (Murat et al, 2014) but not in vivo and it does not affect NCL binding (Lista et al, 2017b; Martins et al, 2018). Such differences are also observed for LANA1. Dabral et al (2019) report that the G4-stabilising ligand TMPyP4 reduces the translation of *LANA1* mRNA, but we show here that PhenDC3 or PhenDH2 treatments increase LANA1 protein expression and the presentation of LANA1-derived antigens. It is, thus, likely that different G4 ligands affect RNA structures differently by being able to either stabilise or destabilise them and/or to interfere with their ability to represent binding platforms for various RNA-binding factors. Considering the dynamic function of the herpes viral G4s, this suggests that G4 structures and activities can be specifically targeted for therapeutic intervention and that screening of G4-targeting compounds should be carried out in an in vivo setting with an intact message to obtain a specific activity.

## Materials and Methods

### Annealing of RNA sequences for CD spectroscopy

The G-rich RNA oligonucleotide (ON) sequences LANA13 (5′-AGGAGGUGGAGGA), genomic loci 2563, G-score 21, and LANA16 (5′-AGGAGCAGGAGGUGGA, genomic loci 2635, G-score 18) (Dabral et al, 2019) were purchased in Eurogentec as lyophilized solid after HPLC separation. They were reconstituted in DEPC water, and their concentration calculated by measuring the UV absorption at 260 nm on a Hitachi 2900 spectrometer. RNA ON buffered solutions (5 μM ON; Li Cacodylate 10 mM, KCl 100 mM) were annealed in a thermo-block by heating 2 min at 95°C and let it cool until reaching room temperature.

### CD footprint

The CD of the previously annealed ON sequences (LANA13 and LANA16) were measured alone or in the presence of 2 M equivalents of PhenDC3 or PhenDH2 in a Jasco J-1500 CD spectrometer using a quartz cuvette of 0.5-cm path length. The parameters were four accumulations, CD scale 200 mdeg/0.1 dOD, scanning speed 100 nm/min. The spectra shown were mathematically processed using the Savitzky–Golay method with 20 points of window (del Villar-Guerra et al, 2017).

### CD melting

The CD melting of the ON sequences containing (LANA13 and LANA16), alone or in the presence of ligand, were monitored using a Jasco J-1500 CD spectrometer equipped with external temperature water-circuit control. The CD was recorded at 262 and 295 nm every 0.2°C change, when heating the samples from 20°C to 95°C at a speed of 0.2°C/min.

### UV melting

The UV melting of the ON sequences containing (LANA13 and LANA16) in absence of ligand were monitored using a Spectro UV Cary-300 equipped with external temperature water-circuit control. The UV was recorded at 295 nm when heating the samples from 20°C to 95°C at a speed of 0.2°C/min.

### Expression plasmids

Putative G4 RNA sequences were identified in the LANA1-encoding and Ova-encoding mRNAs using the QGRS Mapper: https://bioinformatics.ramapo.edu/QGRS/index.php (Kikin et al, 2006).

All expression plasmids were constructed using the pcDNA3 vector and amplified in the *Escherichia coli* DH5α strain. The constructs named Ova, GAr-Ova and Ova-GAr encode the following proteins, respectively: Ovalbumin without the first 50 amino acids, the same protein with the GAr domain from EBNA1 fused to its N terminus or C terminus (Yin, 2003).

The pA3F-LANA1 plasmid carrying the Flag-tagged ORF73 of KSHV in pcDNA3 vector, which encodes wild-type LANA1 protein, was a kind gift from Pr. Blondel (University of Brest). LANA1-SL8 construct was created by inserting the SL8 (SIINFEKL peptide from chicken ovalbumin) coding sequence after the C-terminal domain of LANA1. LANA1ΔCR-SL8 construct was generated using the QuikChange Site-Directed Mutagenesis System (Stratagene) and the construct LANA1-SL8 as template.

The plasmids c-myc_IRES_Ova, c-myc_IRES_GAr-Ova, HCV_IRES_Ova, and HCV_IRES_GAr-Ova encode Ovalbumin or a fusion of the GAr domain to the N terminus of ovalbumin, with either the *c-myc* IRES or the *HCV* IRES in their 5′ UTR (Apcher et al, 2009). *HCV* and *c-myc* IRES were amplified from these constructions and fused to the 5′ UTR or 3′ UTR of LANA1-SL8 plasmid to produce the 5′ c-myc-IRES-LANA1, 3′ c-myc-IRES-LANA1, 5′ HCV-IRES-LANA1, and 3′ HCV-IRES-LANA1 constructs. The list of primers, cloning method, and resulting plasmids is provided in Table S1.

Table S1 Primers and constructs used for cloning.

### Cell culture

The human lung carcinoma cell line H1299 and the EBV-infected B cell line B95.8 were cultured in RPMI 1640 medium supplemented with 10% FBS, 2 mM L-glutamine, 100 U/ml penicillin and 100 μg/ml streptomycin. KSHV-infected BCP-1 cell line was cultured in RPMI medium containing 10% FBS, 4 mM L-glutamine, 100 U/ml penicillin, 100 μg/ml streptomycin, and 5 mM Hepes. All cells were cultured at 37°C in a humidified 5% CO_2_ incubator.

### Transfection and drug treatment

H1299 cells were seeded in six-well plates (7 × 10^4^ cells/well) and transient transfections were carried out 20–24 h later using the Genejuice reagent (Merck Bioscience) according to the manufacturer’s protocol. For cell treatments with PhenDC3, PhenDH2, and PDS (Lista et al, 2017b; Reznichenko et al, 2019), cells were incubated with 2 μM of drug 24 h after transfection. Drug stock solutions were prepared in DMSO (Euromedex).

### Western Blotting

40 h after transfection, cells were resuspended in lysis buffer (20 mM Hepes KOH, pH 7.5, 50 mM β-glycero-phosphate, 1 mM EDTA, pH 8, 1 mM EGTA, pH 8, 0.5 mM Na_3_VO_4_, 100 mM KCl, 10% Glycerol, and 1% Triton X-100) supplemented with complete protease inhibitor cocktail (Roche) and protein concentration was measured using a Bradford assay. Samples were separated by electrophoresis in Bolt Bis-Tris Plus gels 4–12% (Invitrogen/Thermo Fisher Scientific), and then transferred onto nitrocellulose membrane (BioTrace NT, Pall Life Science). Membranes were blocked for 1 h in 5% non-fat dry milk in Tris-buffered saline, pH 7.6, containing 0.1% Tween-20. Proteins were probed by overnight incubation at 4°C with the following antibodies: anti-Ovalbumin whole serum (C6534; Sigma-Aldrich), anti-β-actin mouse monoclonal antibody (clone AC-15; Sigma-Aldrich), anti-NCL polyclonal rabbit antibody (ref 22758; Abcam), anti-Flag mouse monoclonal antibody (F3165; Sigma-Aldrich), anti-EBNA1 mouse monoclonal antibody OT1X (Cyto-Barr BV), and anti-LANA1 (KSHV ORF73) mouse monoclonal antibody (D325-3; MBL International). Membranes were then incubated with appropriate HRP-conjugated secondary antibodies (Dako) and detection of immune complexes was performed using Pierce ECL, WestDura, or West Femto (Thermo Fisher Scientific) and myECL Imager (Thermo Fisher Scientific). Protein bands were quantified by densitometry analysis in Fiji/ImageJ (Schneider et al, 2012), using actin bands for normalisation.

Predicted molecular weight for the full-length LANA1 is 135 kD; however, as it was previously described, the LANA1 full-length protein migrate at 234 kD in SDS–PAGE (Rainbow et al, 1997). In addition, the LANA1ΔCR product is predicted to have a molecular weight of 61 kD, but migration with SDS–PAGE resulted in an observed band of 100 kD.

### RNA FiSH

H1299 cells were seeded in 24-well plates (2 × 10^4^ cells/well) and transient transfections were carried out 20–24 h later using the Genejuice reagent (Merck Bioscience) according to the manufacturer’s protocol. 24 h after transfection or after drug treatment, the cells were briefly washed with ice-cold PBS, fixed with 4% PFA during 20 min at room temperature, and washed again with PBS. Cells were then incubated in 70% ethanol for 4–24 h at 4°C, after intermediate dehydrating steps using 30% and 50% ethanol. For rehydration, the cells were incubated in 50% and 30% ethanol and further washed with PBS. Subsequently, cells were permeabilised with PBS 0.4% Triton 0.05% CHAPS for 5 min at room temperature. After three PBS washes, samples were pre-treated with two subsequent incubations in FiSH wash buffer (10% formamide and 2X SSC in ddH_2_O) for 10 min and then in FiSH hybridisation buffer (10% formamide, 2X SSC, 2 mg/ml BSA, 0.2 mg/ml *E. coli* tRNA, 0.2 mg/ml sheared salmon sperm DNA) for 30 min at room temperature. Coverslips were then incubated overnight in a wet chamber at 37°C in FiSH hybridisation buffer supplemented with 10% dextran sulphate and 100 nM of FiSH Stellaris probes targeting *Ovalbumin* or *LANA1* mRNAs (Biosearch Technologies). Coverslips were washed twice 20 min in FiSH hybridisation buffer and 5 min in FiSH Wash buffer and subsequently stained with DAPI. Samples were mounted using Dako mounting solution (Dako) and observed with a LSM 800 (Zeiss) confocal laser microscope. Images were obtained using Zen software (Zeiss) and the Costes colocalisation factor between nuclei (DAPI channel) and targeted mRNAs (Cy3 channel), called Correlation_Costes_DAPI_Cy3, were obtained using the software CellProfiler (McQuin et al, 2018), and later used for statistical analysis.

### Proximity ligation assay (RNA-protein)

Cells were cultured, fixed, permeabilised, and submitted to a pre-hybridisation step as described above. For target-mRNAs hybridisations, DNA probes were denatured for 5 min at 80°C and diluted in hybridisation buffer at a concentration of 50 ng of probes per sample. The probes 5′ GCAGCAGACTACACCTCCACACTCACC-biotin 3′ and 5′-CTGCTTCATTGATTTCTGCATGTGCTGCATGGACAGCTTGAAAAA-digoxigenin 3′ were designed to target *LANA1* and *Ovalbumin* mRNAs, respectively. Samples were overnight incubated with the denatured DNA probes in a wet chamber at 37°C. Samples were then washed in hybridisation buffer, FiSH wash buffer, and PBS and saturated with PBS 3% BSA 0.1% saponin for 1 h at room temperature. Primary antibodies were diluted in the saturation solution, kept for 30 min at room temperature and then overnight incubated with tested samples at 4°C in a wet chamber. The following antibodies were used to perform the PLA: anti-Digoxigenin mouse monoclonal antibody (clone DI-22, D8156; Sigma-Aldrich), anti-Biotin mouse monoclonal (clone BN-34, B7653; Sigma-Aldrich), and anti-NCL rabbit polyclonal antibody (ab22758; Abcam). Afterwards, PLA was performed using Duolink PLA kit and plus/minus probes (Sigma-Aldrich) following the manufacturer’s instructions. Coverslips were finally mounted using Dako mounting buffer (Dako) after nuclear staining with DAPI and observed using an LSM 800 confocal laser microscope. Images were obtained using the Zen software and analysed using Fiji/ImageJ.

### Antigen presentation

Naïve OVA257-264-specific CD8^+^ T-cells were isolated from the peripheral and mesenteric lymph nodes of OT-I-mice using the CD8^+^-isolation kit (Miltenyi Biotec). Subsequently, 4 × 10^5^ CD8^+^ T-cells were co-cultured with 10^5^ H1299 cells previously transfected with the indicated constructs and a mouse Kb expression vector (a kind gift from C Watts, University of Dundee) and treated or not with the tested drugs. Cells were cultured in RMPI 1640 (Thermo Fisher Scientific) supplemented with 2 mM L-glutamine, 100 U/ml streptomycin and 100 U/ml penicillin (Invitrogen), 10% FBS, 0.05 mM 2-mercaptoethanol, and 5 mM Hepes (Sigma-Aldrich) for 3 d at 37°C with 5% CO_2_. Supernatants were collected from the co-cultures and IL-2 levels were measured by ELISA using ELISA MAX Standard kit (BioLegend) following the manufacturer’s protocol. Signals were measured using a FLUOstar Optima (BMG Labtech) and data were analysed using the software Optima Control v2.20R2.

### RNA extraction, RT-qPCR, and RNA co-IP assay

Transfected cells were washed in cold PBS and total RNA extraction was performed using RNAeasy mini Kit (QIAGEN), following the manufacturer’s instructions. cDNA synthesis was carried out using the Moloney murine leukaemia virus M-MLV reverse transcriptase and Oligo(dT)_12-18_ primer (Life technologies). qPCR was performed using the StepOne real-time PCR system (Applied Biosystems) with Perfecta SYBR Green FastMix (Quanta Biosciences) and the following primers (custom primers by Invitrogen): Actin Forward 5′-TCACCCACACTGTGCCCATCTACGA-3′, Actin Reverse 5′-TGAGGTAGTCAGTCAGGTCCCG-3′, LANA1 Forward: 5′-ATCTCCTGCATTGCCACCCACGC-3′, LANA1 Reverse: 5′-TCTCAGGCTACGCAGGGTAGACG-3′, Ova Forward 5′-GCAAACCTGTGCAGATGATG-3′, Ova Reverse 5′-CTGCTCAAGGCCTGAGACTT-3′. In vitro RNA co-IP was carried out as described elsewhere (Candeias et al, 2008). Briefly, 1 μg of total RNA extracted from cells was co-incubated under agitation with 100 ng of recombinant NCL (provided by Dr. Teulade-Fichou) in the binding buffer (50 mM Tris, pH 7.5, 150 mM NaCl, 0.02 mg/ml yeast tRNA, and 0.2 mg/ml BSA) for 15 min at 37°C. After incubation, NCL–RNA complexes were pulled down at 4°C using G-coated sepharose beads (Sigma-Aldrich) with an anti-NCL rabbit polyclonal antibodies (ab22758; Abcam) according to standard conditions and purified using the TRIzol (Life Technologies). Precipitated RNAs were then analysed by RT-qPCR.

### Statistical analysis

Data were analysed by unpaired Mann–Whitney’s test or *t* test on GraphPad Prism 9. For Western blot, PLA dot counts, and qPCR analyses, represented data are the mean and the SD of a minimum of three independent experiments. For RNA co-IP analyses, represented data are the mean and the standard error of a minimum of three independent experiments. For FiSH analysis, data represented on graphs are the median value, 25% and 75% quartiles delimiting the interquartile range, the maximum and minimum values and the outliers. In all cases, *P* > 0.1234 (ns), *P* < 0.0332 (*), *P* < 0.0021(**), and *P* < 0.0002(***).

## Supplementary Material

Reviewer comments
