# Supplementary material for: The different activities of RNA G-quadruplex structures are controlled by flanking sequences
Source: Life Sci Alliance. 2021 Nov 16;5(2):e202101232. doi: 10.26508/lsa.202101232 (PMC8605322; doi:10.26508/lsa.202101232)
Supplement: Supplementary file 1 [file LSA-2021-01232_TableS1.docx]

| **Generated Construct** | **Template** | **Cloning method** | **Sequence (5’-3’)** |
| --- | --- | --- | --- |
| **LANA1-SL8** | LANA1 -  pcDNA3 | Side Directed Mutagenesis | CTGGGGACTCTCCACAGGAAATGACAAGTATAATCAACTTTGAAAAACTG GGGATATCCATCACACTGGCGGCCGCT AGCGGCCGCCAGTGTGATGGATATCCCCAGTTTTTCAAAGTTGATTATAC TTGTCATTTCCTGTGGAGAGTCCCCAG |
| **LANA1-N-**  **terminal** | LANA1-SL8 | PCR,  restriction ligation | GCATAAGCTTATGGCGCCCCCGGGAATGCGC CGCGGAATTCCTGACTTTCCTTGCTAATCTCGTTGT |
| **LANA1-C-**  **terminal- SL8** | LANA1-SL8 | PCR,  restriction ligation | GCGCGAATTCATCTTGCACGGGTCGTCATCCGAGGACG CGCGGGGCCCTTACTTGTCGTCATCGTCTTTGTAGTCC |
| **LANA1ΔCR** | LANA1-N-  terminal, LANA1-C-  terminal- SL8 | restriction ligation | - |
| **5’ c-myc-IRES- LANA1** | c-myc-Ova (41) | PCR,  restriction ligation | GCGC AAGCTT AACTCGCTGTAGTAATTCCAGCG GCGCGGATCCTGGGTTTACTCTTCCCG |
| **3’ c-myc-IRES- LANA1** | c-myc-Ova (41) | PCR,  restriction ligation | GCGCTCTAGATCCCCTGTGAGGAACTACTGT CGTAGGGCCCGATGCACGGTCTACGAGACCT |
| **5’ HCV-IRES- LANA1** | HCV-Ova (41) | PCR,  restriction ligation | GCGC AAGCTT TCCCCTGTGAGGAACTACTGT CGTAGGATCCAGGTCTCGTAGACCGTGCATC |
| **3’ HCV-IRES- LANA1** | HCV-Ova (41) | PCR,  restriction ligation | GCGCTCTAGAAACTCGCTGTAGTAATTCCAGCG GCGCGGGCCCTGGGTTTACTCTTCCCG |

**Supplementary Table 1**. Primers and constructs used for cloning
